# Correlation Between Coronary Artery Calcium Score and Triglyceride-Glucose Index in Post-menopausal Women

**DOI:** 10.7759/cureus.39034

**Published:** 2023-05-15

**Authors:** Dogac Caglar Gurbuz, Eser Varis

**Affiliations:** 1 Department of Cardiology, Koç University, Istanbul, TUR; 2 Department of Cardiology, Private Istanbul Hospital, Istanbul, TUR

**Keywords:** coronary artery calcium score, triglyceride-glucose index, computed tomography angiography, post-menopausal women, tyg index, coronary artery disease, cacs

## Abstract

Introduction: To clarify the correlation between coronary artery calcium score (CACS) and triglyceride-glucose (TyG) index in post-menopausal women.

Methods: Post-menopausal women who underwent computed tomography angiography with the suspicion of acute coronary syndrome were included in the study. Patients were categorized into three groups (CACS < 100 as group 1, CACS = 100-300 as group 2, and CACS > 300 as group 3). Groups were compared with regard to demographic characteristics, laboratory test outcomes, electrocardiogram findings, and the TyG index.

Results: The study was conducted by examining the data of 228 patients. Median TyG index was 9.0 and median CACS was 79.5. The median age was significantly lower in group 1 (p = 0.001). Diabetes mellitus rate and smoking rate were higher in group 3 compared to the other groups (p = 0.037 and p = 0.032). The glucose level was significantly higher for group 3 (p = 0.001). Additionally, the TyG index was 9.3 in group 3 and was statistically significantly higher than the values in group 1 and group 2 (8.9 and 9.1, respectively) (p = 0.005). There was a moderate correlation between CACS and age (correlation coefficient (CC): 0.241, p = 0.001). Also, there was a significant correlation between glucose level and CACS (CC: 0.307, p = 0.001). A high correlation was found between the TyG index and CACS (CC: 0.424, p = 0.001).

Conclusion: Our study demonstrated for the first time that there was a strong correlation between the TyG index and CACS in post-menopausal patients. In addition, patients with increased age, patients with higher glucose levels, and diabetic patients had significantly higher CACS.

## Introduction

Cardiac diseases are the most common cause of mortality worldwide, and coronary artery disease (CAD) causes almost half of these deaths [1]. Scoring systems were developed to prevent CAD-related morbidity and mortality, assess patients according to risk stratification, and predict treatment outcomes. Coronary artery calcium score (CACS) simply calculates the amount of calcified plaque in coronary arteries. Previous studies found significant correlations between CACS with myocardial infarction, complications during cardiac interventions, cardiac mortality, and all-cause mortality [2]. Moreover, the American College of Cardiology Foundation/American Heart Association consensus stated that higher CACS increased major cardiovascular risk, even in asymptomatic patients [3]. Despite the benefits of CACS, evaluation of CACS has some negative aspects, including requirements for adequate quality computer tomography and a trained radiologist, radiation exposure, and increased cost.

The triglyceride-glucose (TyG) index was developed by Simental-Mendia et al. The TyG index is accepted as a new marker to clarify the presence of insulin resistance, especially in patients with metabolic syndrome. Previous reports emphasized the strong correlation between metabolic syndrome and CAD [4]. Wen and colleagues analyzed the impact of TyG levels on cardiovascular diseases, and the authors concluded that a TyG index higher than 9.0 had predictive value for major cardiovascular events [5]. In another study by Park et al., which evaluated the relationship between the TyG index and diabetes mellitus, they found significantly increased coronary artery stenosis risk in patients with a higher TyG index [6]. Also, post-menopausal women have a higher risk of CAD [7].

Although previous papers evaluated the role of the TyG index for CAD, to our knowledge, no study has evaluated the relationship between CACS and TyG index in post-menopausal women. In the present study, we aimed for the first time to clarify the correlation between CACS and TyG index in post-menopausal women.

## Materials and methods

The data for patients who presented to a tertiary healthcare institution emergency service and cardiology outpatient clinic with a complaint of chest pain were evaluated prospectively. Post-menopausal women who underwent computed tomography angiography (CTA) with the suspicion of acute coronary syndrome were included in the study. Menopausal status was evaluated with patient history and hormonal evaluation of patients. The study was carried out between January 1, 2021, and January 1, 2023, after approval of the local ethics committee. Demographic data, comorbidities, and cardiac and non-cardiac medical histories of the patients were recorded. Patients with suspected non-cardiac chest pain (pneumonia, trauma) and patients not suitable for contrast-enhanced imaging (chronic kidney disease, contrast allergy, etc.) were excluded from the study. In addition, patients with increased cardiac markers, patients with unstable hemodynamics, and patients with missing data were not included in the study.

Patients with chest pain were first evaluated with electrocardiogram and cardiac marker values. Patients not at high risk of acute myocardial infarction were evaluated with CTA. CTA imaging was performed without the use of contrast in the first stage, and then it was performed with 80-120 mL IV injection of contrast material. Oral metoprolol was given to patients with heart rates above 70 beats/minute to improve image quality. After sufficient scanning delay, an electrocardiogram-gated image was obtained from the pulmonary artery bifurcation to the inferior heart border. Evaluation of stenosis and calculation of CACS score were evaluated by a single radiologist experienced in CTA. All patient blood samples were analyzed in the same laboratory. TyG index was calculated with the following formula: Ln (fasting triglycerides (mg/dL) × fasting glucose (mg/dL)/2).

Patients were categorized into three groups: CACS < 100 as group 1, CACS = 100-300 as group 2, and CACS > 300 as group 3. Groups were compared with regard to demographic characteristics, laboratory test outcomes, electrocardiogram findings, and the TyG index.

Statistical analysis

Statistical analysis was performed with the Statistical Package for the Social Sciences (SPSS) version 26 (IBM Corp., Armonk, NY) program. The normality test was applied with the Kolmogorov-Smirnov test and Q-Q plots. Continuous variables were compared using the Kruskal-Wallis test, and Tamhane's T2 test was used for post-hoc analysis. Quantitative data are shown as median (interquartile range). Categorical parameters were compared with the χ2 test and shown as n (%). Correlations between CACS, age, and TyG index were evaluated with Spearman's rank correlation coefficient. Multivariate analysis was performed on the factors that exhibited a statistically significant association with high CACS values in the univariate analysis. P-values of less than 0.05 were accepted as statistically significant.

## Results

The study was conducted by examining the data of 228 patients. The median age of the patients was 54.0 years, and the median BMI was 26.0 kg/m^2^. A total of 130 (57.0%) patients were diagnosed with hypertension, and 57 (25.0%) patients had diabetes mellitus. The rate of smokers was 38.2%. The median TyG index was 9.0 and the median CACS was 79.5. There were no abnormal ECG findings in 205 (89.9%) patients. Early repolarization was present in 14 (6.1%) patients, previous ischemia findings were noted in four (1.8%) patients, and newly developed ischemia findings were in five (2.2%) patients (Table 1). Multivariate analysis was conducted on the factors of age, presence of diabetes mellitus, smoking, glucose value, and TyG value, which were identified as being associated with high CACS values in the univariate analysis. The results of the multivariate analysis revealed a statistically significant relationship between high CACS values and age, presence of diabetes mellitus, glucose level, and TyG value (p = 0.001, p = 0.023, p = 0.044, and p = 0.028, respectively).

**Table 1:** Demographic characteristics and laboratory values of all patients * Median (interquartile range); BMI: body mass index; HDL: high-density lipoprotein; LDL: low-density lipoprotein; TyG: triglyceride-glucose; CACS: coronary artery calcium score; ECG: electrocardiogram; HbA1c: glycosylated hemoglobin.

	n = 228
Age (years)*	64.0 (49.0-74.0)
BMI (kg/m²)*	26.0 (21.0-33.0)
Hypertension, n (%)	130 (57.0%)
Diabetes mellitus, n (%)	57 (25.0%)
Smoking, n (%)	87 (38.2%)
HbA1c (%)*	6.5 (5.0-7.9)
Total cholesterol (mg/dl)*	198.0 (167.0-228.0)
HDL (mg/dl)*	39.0 (34.0-44.0)
LDL (mg/dl)*	189.0 (160.0-223.8)
Triglycerides (mg/dl)*	161.0 (122.0-207.0)
Glucose (mg/dl)*	102.0 (88.0-118.0)
TyG index*	9.0 (8.7-9.3)
CACS*	79.5 (42.0-198.5)
ECG findings	
Normal	205 (89.9%)
Early repolarization	14 (6.1%)
Previous ischemia findings	4 (1.8%)
Ischemia findings	5 (2.2%)

A comparison of patient demographic data and laboratory values is shown in Table 2. Patients were divided into three groups as follows: CACS < 100 (group 1), CACS = 100-300 (group 2), and CACS > 300 (group 3). Median CACS was 49.0 in group 1, 197.0 in group 2, and 779.5 in group 3. The median age was significantly lower in group 1 (53.0) compared to group 2 (56.0) and group 3 (55.5) (p = 0.001). The median body mass index was found to be similar between the groups (p = 0.310). There was no difference between the groups in terms of hypertension rates (p = 0.204), but the diabetes mellitus rate was higher in group 3 compared to the other groups (p = 0.037). The smoking rate was 66.7% in group 3 and this was statistically significantly higher than the other groups (p = 0.032). Lipid profile parameters did not differ significantly between the groups. However, the glucose level was significantly higher for group 3 (p = 0.001). Additionally, the TyG index was 9.3 in group 3 and was statistically significantly higher than the values in group 1 and group 2 (8.9 and 9.1, respectively) (p = 0.005).

**Table 2:** Comparison of patient demographic data and laboratory values according to coronary artery calcium score group * Median (interquartile range); BMI: body mass index; HDL: high-density lipoprotein; LDL: low-density lipoprotein; TyG: triglyceride-glucose; CACS: coronary artery calcium score; ECG: electrocardiogram; HbA1c: glycosylated hemoglobin. Lowercase letters define the group that makes the difference. Different letters (such as a and b) show that there is a difference.

	CACS < 100	CACS = 100-300	CACS > 300	p-value
Number of patients	131 (57.5%)	79 (34.6%)	18 (7.9%)	
Age (years)*	53.0 (47.0-57.0)^a^	56.0 (51.0-63.0)^b^	55.5 (51.8-64.3)^b^	0.001
BMI (kg/m²)*	26.0 (22.0-33.0)	27.0 (21.0-34.0)	27.5 (24.8-33.3)	0.310
Hypertension, n (%)	77 (58.8%)	40 (50.6%)	13 (72.2%)	0.204
Diabetes mellitus, n (%)	29 (22.1%)^a^	19 (24.1%)^a^	9 (50.0%)^b^	0.037
Smoking, n (%)	48 (36.6%)^a^	27 (34.2%)^a^	12 (66.7%)^b^	0.032
HbA1c (%)*	6.4 (5.2-7.6)	6.5 (4.9-8.2)	6.5 (4.7-7.7)	0.888
Total cholesterol (mg/dl)*	198.0 (167.0-229.0)	188.0 (167.0-228.0)	205.5 (179.3-225.0)	0.849
HDL (mg/dl)*	38.0 (34.0-44.0)	38.0 (34.0-44.0)	42.5 (34.8-45.8)	0.333
LDL (mg/dl)*	184.0 (160.0-219.0)	190.0 (162.0-224.0)	197.5 (151.3-235.5)	0.763
Triglycerides (mg/dl)*	137.0 (118.0-200.0)	167.0 (125.0-215.0)	189.0 (152.5-219.3)	0.078
Glucose (mg/dl)*	102.0 (88.0-116.0)	103.0 (92.0-121.0)	122.5 (101.5-143.3)	0.001
TyG Index*	8.9 (8.6-9.2)^a^	9.1 (8.8-9.3)^a^	9.3 (8.9-9.6)^b^	0.005
CACS	49.0 (24.0-72.0)^a^	197.0 (141.0-261.0)^b^	779.5 (452.5-879.8)^c^	0.001

The correlation between CACS, age, and TyG index is shown in Table 3. There was a low correlation between CACS and age (correlation coefficient (CC): 0.241, p = 0.001). Also, there was a moderate correlation between glucose level and CACS (CC: 0.307, p = 0.001). A moderate correlation was found between the TyG index and CACS (CC: 0.424, p = 0.001).

**Table 3:** Correlation between coronary artery calcium score with age, glucose, and TyG index TyG: triglyceride-glucose; CACS: coronary artery calcium score.

CACS	Age	Glucose	TyG index
Correlation coefficient	0.241	0.307	0.424
P-value	0.001	0.001	0.001

Multivariate analysis was conducted on the factors of age, presence of diabetes mellitus, smoking, glucose value, and TyG value, which were identified as being associated with high CACS values in the univariate analysis. The results of the multivariate analysis revealed a statistically significant relationship between high CACS values and age, presence of diabetes mellitus, glucose level, and TyG value (p = 0.001, p = 0.023, p = 0.044, and p = 0.028, respectively) (Table 4).

**Table 4:** Multivariate analysis was conducted to evaluate the factors associated with high CACS values CACS: coronary artery calcium score; CI: confidence interval; TyG: triglyceride-glucose.

	Odds ratio	95% CI	p-value
Age	1.115	1.062-1.171	0.001
Diabetes mellitus	2.165	1.113-4.213	0.023
Smoking	1.166	0.634-2.144	0.620
Glucose	1.047	1.015-1.101	0.044
TyG index	3.763	1.965-8.909	0.028

## Discussion

CAD is being diagnosed with increasing frequency, and it is critical to identify methods that will enable early diagnosis of CAD, determine the optimal treatment modality, and predict the success of treatment. The correlation of CACS with CAD was proven by many studies; however, radiation exposure, cost, and requiring quality equipment and trained personnel are the main limitations of CACS assessment [2,3]. A relatively new diagnostic tool, the TyG index, is used for the diagnosis of metabolic syndrome, diabetes mellitus, and the presence of insulin resistance. Thus, we conducted a prospective study to clarify the correlation between the TyG index and CACS in post-menopausal women. In the present study, we found a strong correlation between the TyG index and CACS in post-menopausal women for the first time. Moreover, our finding revealed that the presence of increased glucose level, increased TyG index, and age were associated with higher CACS in post-menopausal women.

The TyG index is an indirect sign of insulin resistance, and previous research evaluated TyG index levels in many conditions, which could be related to insulin resistance, such as diabetes mellitus, hypercholesterolemia, and erectile dysfunction. Because of the TyG index calculation formula, increments in triglyceride and fasting glucose levels directly increase the TyG index value. Ding et al. reviewed eight cohort studies about the TyG index and cardiovascular diseases, and the authors found that a higher TyG index was associated with atherosclerotic cardiovascular diseases, CAD, and stroke [8]. Pletcher and colleagues found that the presence of diabetes mellitus and high levels of low-density lipoprotein cholesterol were predictive factors for high CACS [9]. In another study by McAvoy et al., patients with higher CACS had a higher risk of atherosclerotic plaque progression and future major cardiovascular event risk [10]. Also, the aforementioned studies show that the factors affecting the TyG index and CACS are similar. In the present study, although cholesterol levels were higher in patients with high CACS scores, the difference was not statically significant according to CACS groups. However, we found a moderate correlation between the TyG index and CACS. According to our results, we believe that the TyG index could be used as an alternative method to CACS in post-menopausal women.

Early diagnosis of atherosclerotic plaques in diabetic patients is crucial due to the high cardiovascular morbidity and mortality of these patients. Lim and colleagues analyzed the impact of glucose levels on atherosclerotic plaque development by analyzing CACS and found that patients with impaired fasting glucose levels and diabetic patients had significantly more severe coronary stenosis, larger atherosclerotic plaque size, and higher CACS [11]. In another study, Giri et al. investigated the impact of glucose levels on coronary arteries by using single-photon emission computed tomography. The authors found significantly more abnormal findings in diabetic patients in comparison with non-diabetic patients. Moreover, Giri et al. claimed that major cardiac events were significantly increased in patients with diabetes mellitus after two years of follow-up, but not in non-diabetic patients [12]. In the present study, increased glucose levels and the presence of diabetes mellitus were found to be predictive factors for higher CACS.

Atherosclerosis is an inevitable process of aging. Newman and colleagues investigated CACS in 614 patients older than 65 years and stated that CACS significantly increased with age [13]. Moreover, Valenti and colleagues evaluated the prognostic value of CACS in the elderly, and age was found to be a predictive factor for higher CACS and higher mortality rates [14]. In another study by Hoff et al., the authors found a 0.5 median score for CACS in patients <40 years old, and a 473 median score for CACS in patients >74 years old [15]. In the present study, we found a low correlation between aging and higher CACS.

Although this study is the first to analyze the correlation between the TyG index and CACS in post-menopausal women, our study has some limitations, including the relatively small patient number and lack of long-term results. Also, the cost of the TyG index and CACS were not compared in the present study, which may be a subject for further studies. In addition, we did not evaluate the duration of some conditions such as diabetes mellitus and hypertension, which may affect the TyG index and CACS. Lastly, we did not focus on the role of the TyG index in clinical practice; we believe that further studies will clarify the role of the TyG index in cardiology practice during the assessment of coronary artery calcification.

## Conclusions

Our study demonstrated for the first time that there was a strong correlation between the TyG index and CACS in post-menopausal patients who were admitted to the cardiology outpatient clinic. In addition, patients with increased age, patients with higher glucose levels, and diabetic patients had significantly higher CACS. The predictive value of the TyG index for the evaluation of coronary artery calcification should be analyzed in further studies.
